# 4D Light-sheet imaging and interactive analysis of cardiac contractility in zebrafish larvae

**DOI:** 10.1063/5.0153214

**Published:** 2023-06-15

**Authors:** Xinyuan Zhang, Milad Almasian, Sohail S. Hassan, Rosemary Jotheesh, Vinay A. Kadam, Austin R. Polk, Alireza Saberigarakani, Aayan Rahat, Jie Yuan, Juhyun Lee, Kelli Carroll, Yichen Ding

**Affiliations:** 1 Department of Bioengineering, Erik Jonsson School of Engineering and Computer Science, The University of Texas at Dallas, Richardson, Texas 75080, USA; 2 Department of Computer Science, Erik Jonsson School of Engineering and Computer Science, The University of Texas at Dallas, Richardson, Texas 75080, USA; 3 Department of Bioengineering, The University of Texas at Arlington, Arlington, Texas 76019, USA; 4 Department of Biology, Austin College, Sherman, Texas 75090, USA; 5 Center for Imaging and Surgical Innovation, The University of Texas at Dallas, Richardson, Texas 75080, USA; 6 Hamon Center for Regenerative Science and Medicine, UT Southwestern Medical Center, Dallas, Texas 75390, USA

## Abstract

Despite ongoing efforts in cardiovascular research, the acquisition of high-resolution and high-speed images for the purpose of assessing cardiac contraction remains challenging. Light-sheet fluorescence microscopy (LSFM) offers superior spatiotemporal resolution and minimal photodamage, providing an indispensable opportunity for the *in vivo* study of cardiac micro-structure and contractile function in zebrafish larvae. To track the myocardial architecture and contractility, we have developed an imaging strategy ranging from LSFM system construction, retrospective synchronization, single cell tracking, to user-directed virtual reality (VR) analysis. Our system enables the four-dimensional (4D) investigation of individual cardiomyocytes across the entire atrium and ventricle during multiple cardiac cycles in a zebrafish larva at the cellular resolution. To enhance the throughput of our model reconstruction and assessment, we have developed a parallel computing-assisted algorithm for 4D synchronization, resulting in a nearly tenfold enhancement of reconstruction efficiency. The machine learning-based nuclei segmentation and VR-based interaction further allow us to quantify cellular dynamics in the myocardium from end-systole to end-diastole. Collectively, our strategy facilitates noninvasive cardiac imaging and user-directed data interpretation with improved efficiency and accuracy, holding great promise to characterize functional changes and regional mechanics at the single cell level during cardiac development and regeneration.

## INTRODUCTION

Cardiac contraction relies on the orchestrated interplay among cells at the tissue level.^1–3^ The continuous investigation of myocardial properties and mechanics is critical for the assessment of cardiac structure and contractility under physiological and pathophysiological conditions. Recent progress has demonstrated that zebrafish is a powerful model for investigating cardiac development and repair due to its optical transparency, genetic tractability, and regenerative capacity.^4–7^ However, conventional techniques face limitations in analyzing cardiac architecture and function of zebrafish heart due to insufficient spatiotemporal resolution and shallow penetration depth. Light-sheet fluorescence microscopy (LSFM) overcomes these challenges by rapidly scanning across the entire ventricle and atrium with high spatiotemporal resolution and minimal photodamage,^8–13^ allowing for the concurrent investigation of myocardial micro-structure and contractile function.

To reconstruct cardiac contraction following LSFM imaging, both prospective^14^ and retrospective^15–19^ synchronization have been developed. Bypassing the extra camera and real-time processing for cardiac gating in the former method, retrospective synchronization allows for the four-dimensional (4D) reconstruction of quasi-periodic cardiac dynamics. However, due to the deluge of raw data and tedious procedures, the retrospective algorithm usually requires over three hours to process ∼100 GB images under the current computational power, limiting our high-throughput capability to iterate and analyze cardiac activities. Since parallelization of algorithms provides an efficient way for the time-consuming computation in biomedical imaging reconstruction,^20^ we designed and implemented our 4D reconstruction algorithm based on parallel computing, resulting in a tenfold improvement in reconstruction efficiency.

In addition, the technical challenge of analyzing intercellular interaction within a complex environment poses a major challenge to assessing cardiac contractility and regional mechanics.^21^ Current unparalleled high-resolution 4D LSFM image stack requires an accurate segmentation approach and a user-directed method to interpret cardiac contraction at the single cell level. In this context, we implemented a deep learning approach termed 3DeeCellTracker^22^ to track individual cells across the entire heart in the zebrafish larva within multiple cardiac cycles, and we developed a virtual reality (VR)-based interactive platform^23–25^ to identify local differences of contracting velocity and quantify cellular motion from end-systole to end-diastole. This further allows us to investigate intricate micro-structure, quantify regional contractility, and assess myocardial mechanics across the ventricle and atrium at different cardiac phases. Collectively, our holistic framework including imaging system construction, parallel computation, and advanced quantitative analysis allows for the *in vivo* investigation of regional variations in myocardium of zebrafish models with high accuracy and efficiency, holding potential to foster our understanding of the cardiac development and regeneration.

## RESULTS

**FIG. 1.**
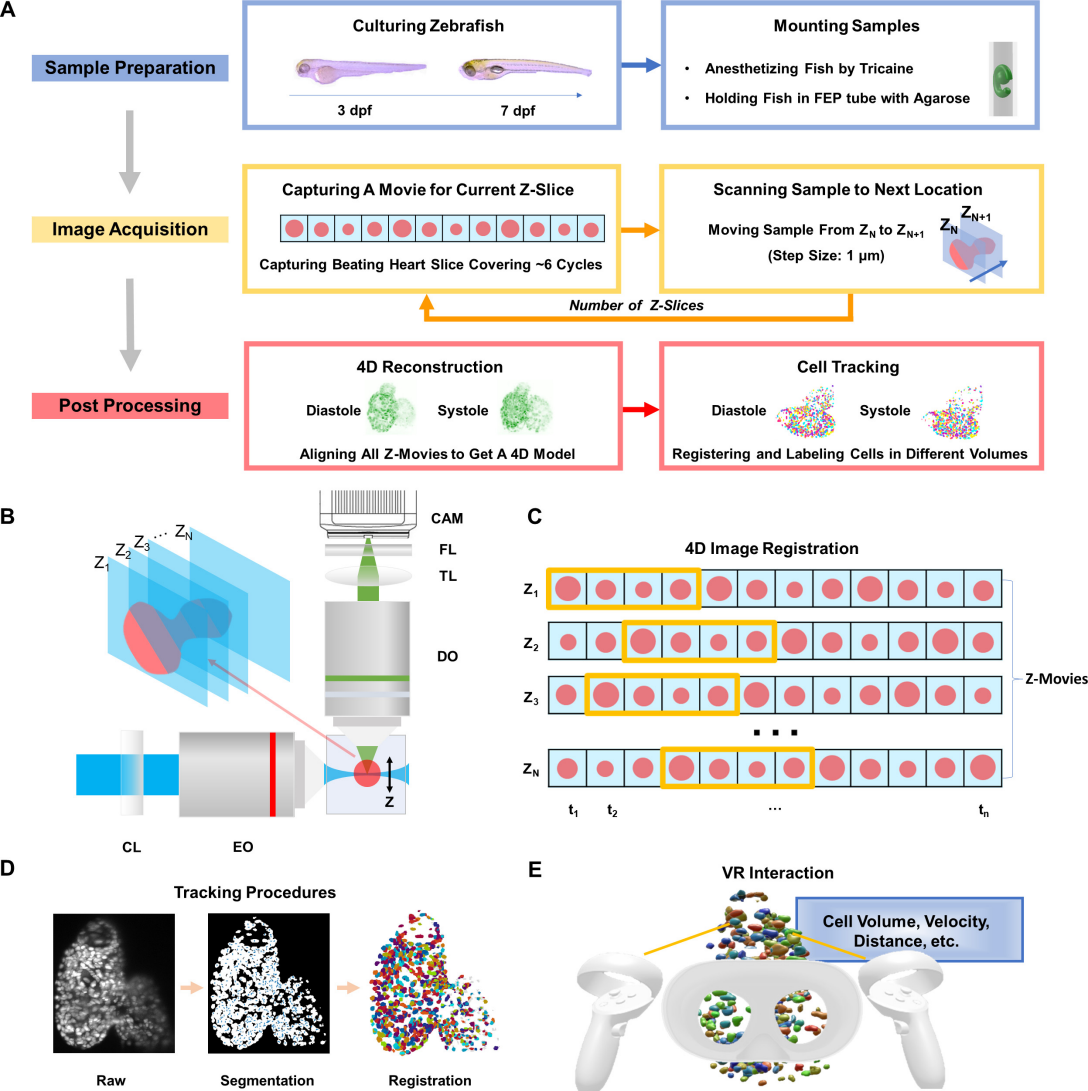
4D light-sheet fluorescence microscopy (LSFM) framework for zebrafish heart imaging and analysis. (a) Workflow of the zebrafish cardiac activity analysis, including major steps from zebrafish preparation in standard E3 medium with phenylthiourea (PTU) and tricaine, to 4D image acquisition, and to reconstruction and single cell tracking. (b) Simplified schematic illustration of the customized LSFM system construction. CL: cylindrical lens. EO: excitation objective. DO: detection objective. TL: tube lens. FL: filter. CAM: sCMOS camera. (c) Illustration of retrospective synchronization for 4D zebrafish image registration. Z-movie indicates a continuous image sequence at a certain depth along the z-axis. Each frame in the image sequence is represented by a red dot, and the starting and ending phases from end-diastole to end-systole are highlighted in the yellow box. (d) Procedures of cell segmentation and tracking. Raw, segmented, and successively registered images are presented from left to right. Individual cells are coded using pseudo-colors. (e) User-directed interaction, including cell selection and quantitative analysis, has been achieved in the virtual environment.

We have developed a framework, including sample preparation, image acquisition, and post-processing [Fig. 1(a) and supplementary material Fig. S1], to demonstrate the feasibility and potential of our prototype for zebrafish heart imaging and analysis. Briefly, we anesthetized zebrafish larvae ranging from 3 to 7 days post fertilization (dpf) with 0.05% tricaine and immobilized them in 0.8% low-melting-point agarose under the microscope, to ensure the growth of the larvae and optimal transparency. Our six-axis translational stage synchronized by the customized LabVIEW control scanned the contracting heart across the sheet of laser, while the sCMOS camera captured image sequences at each slice [Fig. 1(b) and supplementary material video 1]. Our retrospective synchronization algorithm based on image similarity analysis^26^ has been empowered by parallel computation to reconstruct the 4D cardiac contraction [Fig. 1(c)]. We have further tracked individual cells using the established 3DeeCelltracker^22^ within one cardiac cycle and implemented our VR platform for the interactive quantitative analysis of cells across the entire heart [Figs. 1(d) and 1(e)].

## POINT SPREAD FUNCTION CALIBRATION

**FIG. 2.**
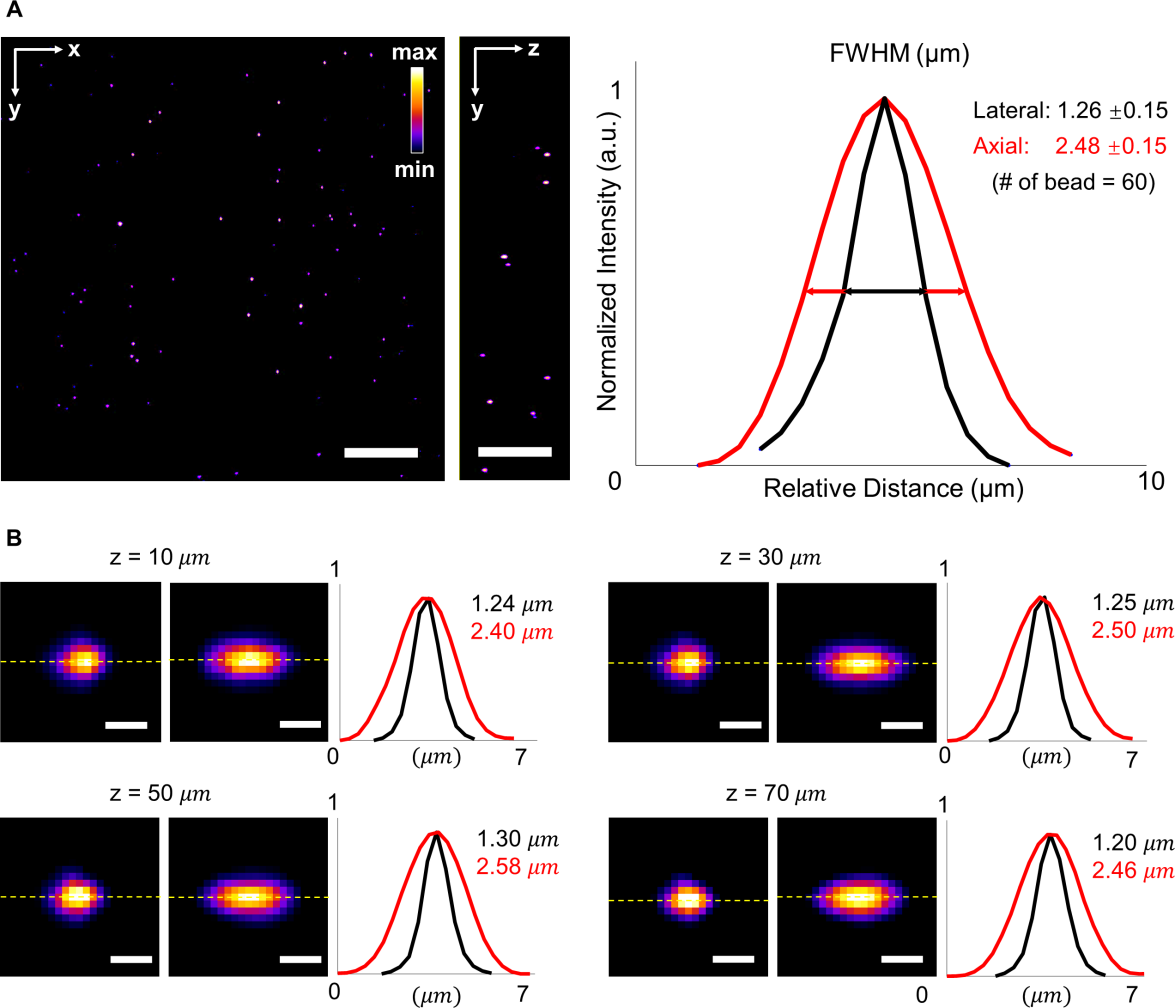
Full width at half maximum (FWHM) of beads captured by LSFM at various depths. (a) Raw data of fluorescent beads in a volume of ∼300 × 300 × 75 *μ*m^3^ in the left panel. The average lateral and axial resolutions of the LSFM are 1.26 ± 0.15 μm and 2.48 ± 0.15 μm, respectively. Normalized intensity is shown in Fire pseudo color. (b) Cross section images of beads at different depths ranging from 0 to 75 *μ*m. Representative lateral and axial resolutions at different depths were measured along the dash lines. Scale bars: (a) 50 and (b) 2 *μ*m.

Our LSFM system is composed of a horizontal detection arm and a cylindrical lens-based illumination (supplementary material Fig. S2). To measure the point spread function (PSF) of our LSFM system, we imaged fluorescent beads with a diameter of ∼0.53 *μ*m as shown by Fire pseudo color [Fig. 2(a), supplementary material video 2). To verify the spatial resolution and minimize the variation of opacity at different depths, we calibrated the PSF across the whole sample and demonstrated the representative results of the full width at half maximum (FWHM) at depths of 10, 30, 50, and 70 *μ*m [Fig. 2(b)]. Our results indicate that the lateral and axial resolutions are 1.26 ± 0.15 and 2.48 ± 0.15 *μ*m (n = 60 beads), respectively, suggesting the invariant spatial resolution within the imaging depth.

## PARALLEL COMPUTATION-BASED RETROSPECTIVE RECONSTRUCTION

**FIG. 3.**
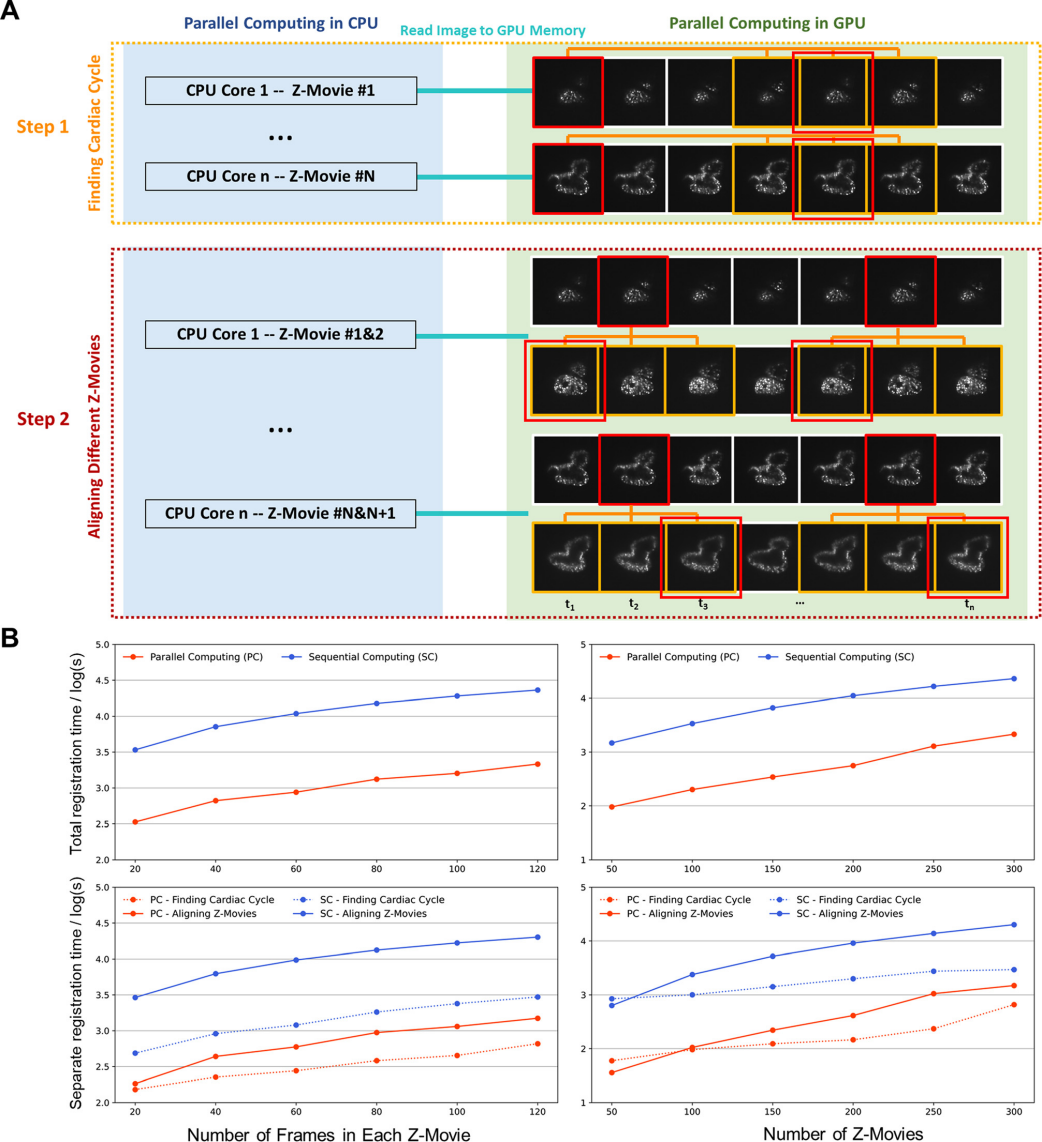
Parallel computation for 4D image registration. (a) Procedures of using parallel computation to register different Z-movies. The initial step was to determine the cardiac cycle. Each CPU core was assigned to initialize the calculation process for one movie, and the GPU was utilized to match a targeted image in red with others in yellow that correspond to the same cardiac phase in subsequent cardiac cycles within the same Z-movie. The matched frame was marked with an additional red box. The next step involved aligning Z-movies in different depths to start from the same cardiac phase. Each CPU core was assigned to initialize the calculation process between two consecutive movies, and the GPU was used to match the targeted image in red with others in yellow that correspond to the same cardiac phase in the next Z-movie. (b) Quantitative comparison of the processing time between parallel computing and sequential computing. The registration time for different numbers of frames (on the left) and movies (on the right) improved by over tenfold in both the overall process (at the top) and in individual steps (at the bottom).

To improve the efficiency of our retrospective synchronization for high-throughput analysis, we have established parallel computation to maximize the usage of multi-core CPU and GPU processors for 4D cardiac reconstruction. In contrast to 3-h processing of 30 000 frames (800 × 800 pixels/frame) from 100 movies, our current method accomplished the same task within 20 min. Briefly, two steps were involved. First, to identify cardiac cycles, we assigned independent Z-movies to individual CPU cores and aligned cardiac phases in different cardiac cycles using GPU cores until all Z-movies were processed [Fig. 3(a)]. Next, to synchronize different Z-movies, we assigned two consecutive Z-movies to each CPU core and aligned cardiac phases within multiple cycles of all movies using GPU cores [Fig. 3(a)]. We implemented image similarity analysis to match the targeted phase (in red) with the potential frames (in yellow) in other cardiac cycles in both steps. Since GPU is able to handle more pixels in parallel,^27^ we tested different numbers of frames and Z-movies to compare the computational efficiency of our new method with the previous version under varying conditions [Fig. 3(b)]. The results indicate that the image registration time is proportional to the number of frames (left panel) and Z-movies (right panel), and the parallel computation improved our reconstruction efficiency over ten times in both steps.

## IMAGING OF THE BEATING ZEBRAFISH HEART

**FIG. 4.**
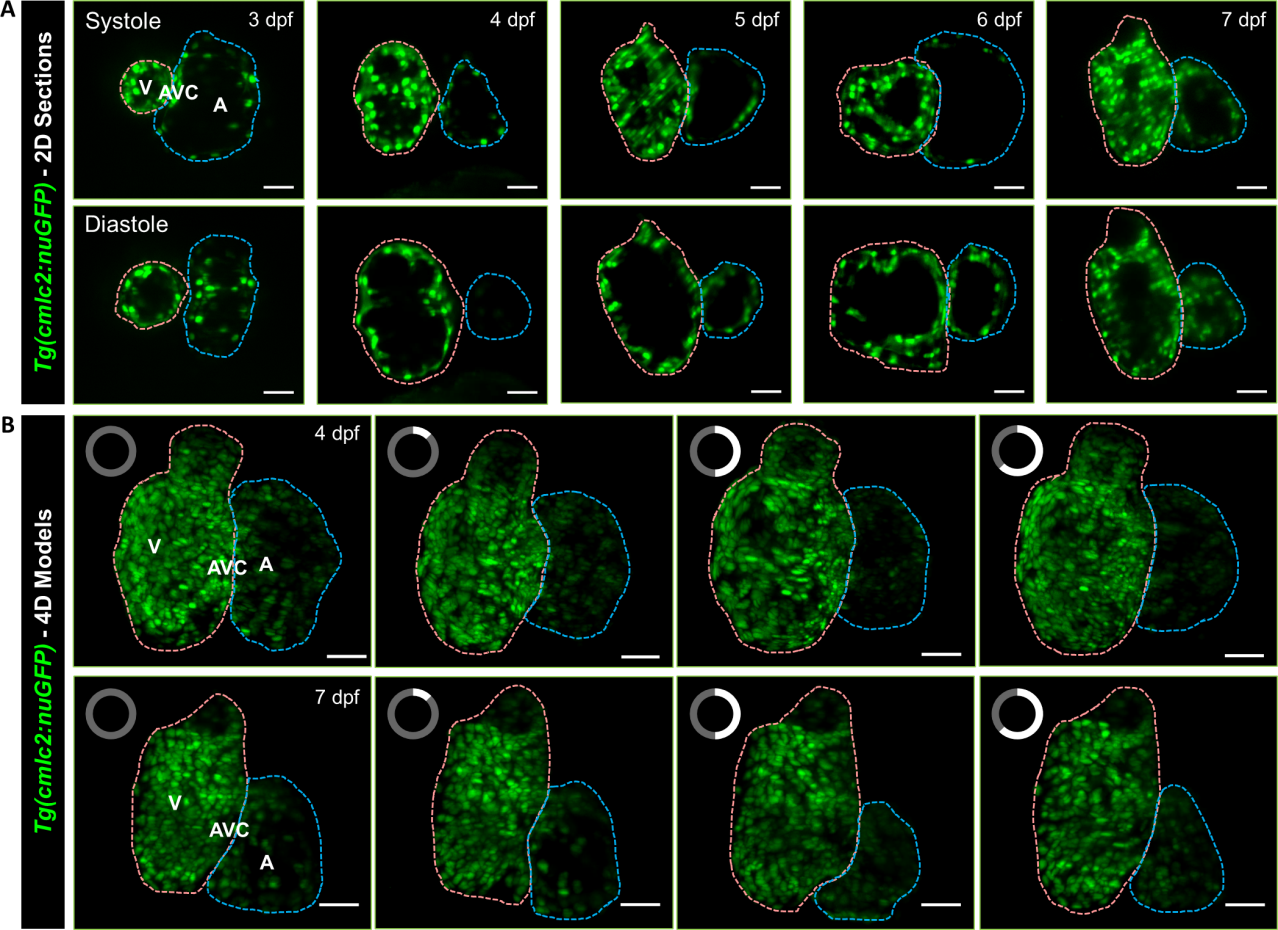
Light-sheet imaging of GFP-labeled cardiomyocyte nuclei in the transgenic *Tg(cmlc2:nucGFP)* zebrafish larvae. (a) Cardiac contraction was captured from ventricular systole to diastole in 3–7 days post fertilization (dpf), respectively. (b) 4D Reconstruction of contracting hearts in zebrafish larvae at 4 (top) and 7 dpf (bottom) was presented as a maximum intensity projection (MIP) image. The clock on the upper left of each frame indicates the cardiac phase starting from end-systole. Cardiac ventricle and atrium are depicted in pink and blue, respectively. V: ventricle. A: atrium. AVC: atrioventricular canal. Scale bar: 30 *μ*m.

We implemented the aforementioned system and algorithm to capture the traveling cardiomyocyte nuclei across the entire ventricle and atrium in transgenic (*Tg(cmlc2:nucGFP))* zebrafish larvae (Fig. 4). We acquired around 100–200 Z-movies per fish (depending on its size) to cover the entire contracting heart at a rate of 200 frames per second (fps), with each movie consisting of 300 frames and the step size of 1 *μ*m between consecutive movies. Each Z-movie usually included five cardiac cycles, but we retained the three complete cardiac cycles during the 4D reconstruction. The LSFM system provided us the single cell resolution to investigate the ventricular and atrial contraction in zebrafish larvae from 3 to 7 dpf [Figs. 4(a) and supplementary material videos 3 and 4), and our reconstruction algorithm allowed us to establish 4D digital heart models from end-systole to end-diastole with 200 volumes per second [Fig. 4(b) and supplementary material videos 5 and 6). The integration of our engineering methods with green fluorescence protein (GFP) labeled myocyte nuclei depicted the contour of the micro-architecture of ventricle (in pink), atrium (in blue), and atrioventricular canal in zebrafish larvae, providing an entry point for the *in vivo* study of myocardial trabeculation and compaction during cardiac development and regeneration.

## TRACKING OF INDIVIDUAL CARDIOMYOCYTE NUCLEI

**FIG. 5.**
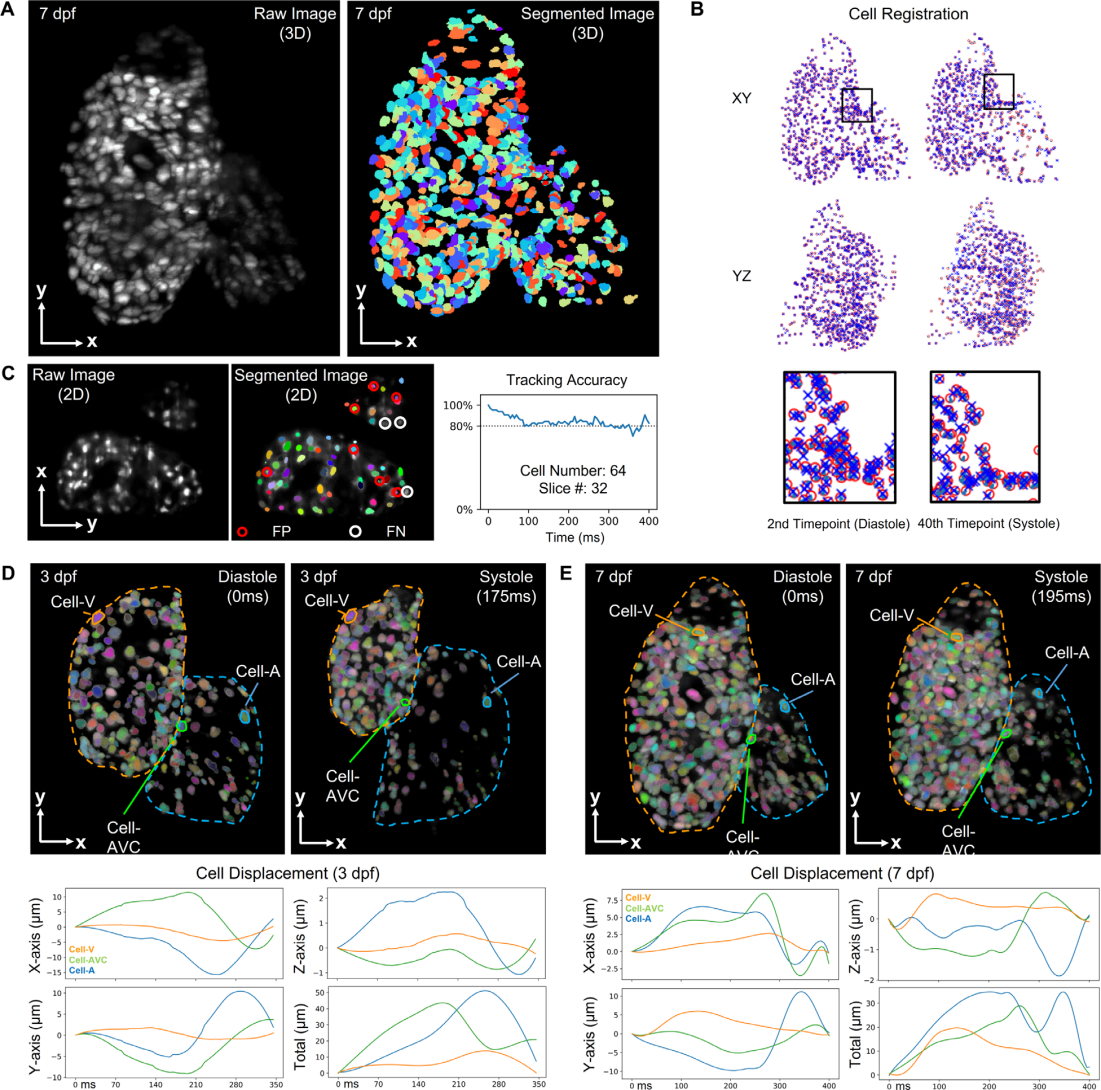
Cell tracking results for zebrafish beating heart. (a) Raw and segmented images of *Tg(cmlc2:nucGFP)* zebrafish heart at 7 dpf were presented as a MIP image. The total number of tracked cells is 580. (b) Cell tracking algorithm enabled us to register cells among various cardiac phases. Manually corrected cells in the first volume were denoted as red circles, while the cells registered by 3DeeCellTracker in target next volume were denoted as blue crosses. (c) Assessment of the tracking accuracy in 64 cells over time. The results indicate false positive (FP) labels where non-cell regions were labeled as cells and false negative (FN) labels where specific cells were not detected. Individual cells were assigned pseudo-colors to differentiate them. (d) and (e) Further displacement analysis allowed for the tracking of representative cells in the ventricle, atrium, and AVC in the 3D space at 3 and 7 dpf, respectively.

To further the quantitative analysis of myocardial contraction and mechanics, we incorporated an established deep learning approach termed 3DeeCellTracker^22^ into our computational framework. Using our reconstructed cardiac LSFM images [Fig. 5(a)], we were allowed to segment and differentiate individual cardiomyocytes within multiple cardiac cycles [Fig. 5(b) and supplementary material videos 7 and 8). The raw images were first segmented and classified into cell or non-cell categories by 3D U-Net, which was followed by the 3D tracking step. To pinpoint the precise cell locations, two approaches, feedforward network and PR-GLS, were utilized. Unlike manual annotation, these methods allowed for efficient registration of cardiomyocytes in current (red circles) and subsequent volumes (blue crosses) within the region of interest, until all volumes were registered. To ensure the accuracy of the tracking results, we selected a 2D slice from the central region of the heart with 64 cell nuclei and manually annotated all cells as the ground truth for comparison [Fig. 5(c)]. Due to the varying intensity and irregular shape of cells close to the boundary, our results indicated that about 80% of cells were correctly tracked using this approach within one cardiac cycle (∼400 ms). To identify differences in contractility across various myocardial regions, we analyzed the displacements of three representative cells in the atrium, ventricle, and atrioventricular canal of zebrafish at 3 and 7 dpf, respectively [Figs. 5(d) and 5(e)]. The results indicated that the atrial cell had the largest displacement during heart contraction, implying that the atrial contractile function undergoes more significant changes compared to other regions within one cardiac cycle.

## USER-DIRECTED ANALYSIS OF CARDIAC CONTRACTION

**FIG. 6.**
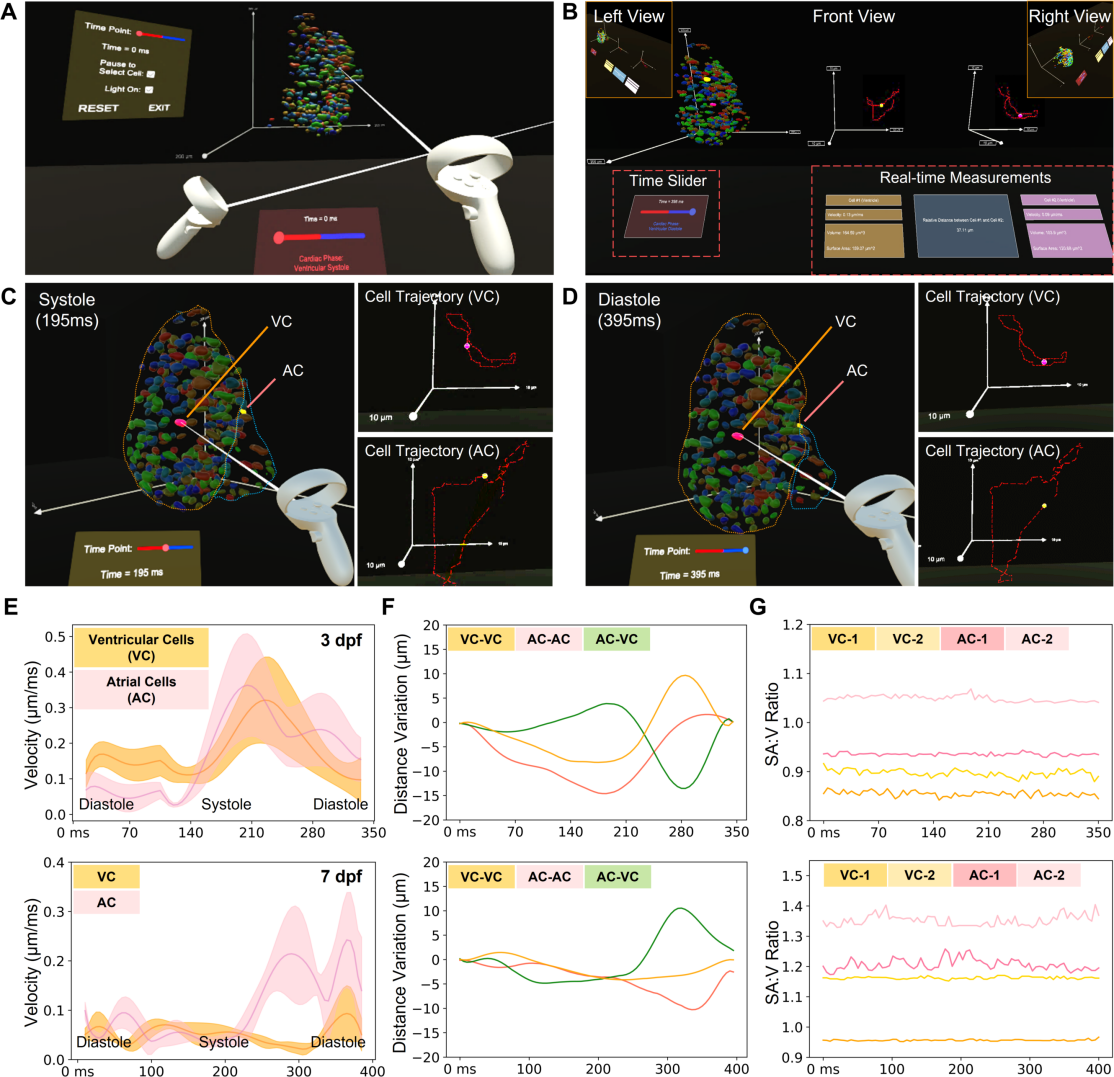
Manipulative functionalities of our VR platform on 4D zebrafish heart models. (a) The VR platform provides users with an immersive viewing and interactive experience of a 4D zebrafish heart model, allowing us to visualize and analyze the heart function in a user-defined mode over time. (b) Overview of the analyzing tool in VR. The available interactions in the VR platform include cell selection, time point selection, time pause, and lighting adjustment. Trajectories for the selected cells are displayed as dotted lines, and a glowing point indicates the current position in the trajectory. After cell selection, the VR platform provides quantitative analysis outputs, including velocity, volume, surface area, and relative distance if two cells are selected. (c) and (d) To validate the platform's functions, we randomly selected two cells in the ventricle and atrium (VC: ventricular cell and AC: atrial cell) and compared their traces at different time points. (e)–(g) After collecting the measurement results from our VR platform, we compared the velocity and relative distance change in one cardiac cycle between selected cells, and the SA:V ratio for selected cells in different heart regions at 3 (top) and 7 dpf (bottom), respectively. (e) The average velocity changes in five ventricular cells and five atrial cells. (f) The relative distance changes between three groups of cells, i.e., two ventricular cells, a ventricular cell and an atrial cell, and two atrial cells. (g) SA:V ratio change of four randomly selected ventricular and atrial cells during one cardiac cycle are depicted over the time course.

Due to numerous traveling cells across the entire heart, an effective data interpretation method could provide an in-depth analysis of cardiac contractile function. We have developed a VR platform that enables us to visualize and assess the regional cardiac contraction with numerous manipulative functionalities, such as selecting arbitrary cells and cardiac phase, and measuring cellular properties (Fig. 6 and supplementary material video 9). To demonstrate the feasibility of our approach, we tracked the whole-cycle trajectories of targeted individual cells and their current positions indicated by glowing points [Figs. 6(a) and 6(b)]. In addition to individual cells, we were able to select two arbitrary cardiomyocytes from different regions, such as ventricle and atrium, to quantify their traveling velocities, volumes, surface areas, and relative distance to each other over time [Figs. 6(c) and 6(d)]. In addition to individual cells, we also could conduct the group analysis to measure average velocity [Fig. 6(e)], relative distance variation between cell pairs [Fig. 6(f)], and surface area to volume (SA:V) ratio of cells [Fig. 6(g)] in different regions within one cardiac cycle for zebrafish at 3 and 7 dpf. Our results reflect that the average velocity of atrial cells was higher than that of ventricular cells, suggesting that the regional contractility of the atrium is always larger. Additionally, the relative distance between cells varies depending on their locations, allowing us to further investigate the local stretch and strain. The SA:V ratio holds promise for evaluating the local strain of individual nuclei and also reflects the developmental change. Collectively, in contrast to conventional pre-defined visualization and operations, we have proved the concept that VR platform holds great potential for multi-dimensional structural and functional investigation of cardiac contraction at the single cell resolution with the user-directed interaction.

## DISCUSSION

Zebrafish is a powerful model for cardiac research due to its optical transparency, remarkable regenerative capacity, and genetic and physiological similarity to humans.^4–7^ We are able to identify over 70% of human genes and more than 80% of human disease genes in at least one zebrafish counterpart.^28^ This makes it an ideal model for both fundamental research and translational investigations in cardiovascular disease. The advent of LSFM fosters the 4D investigation of cardiac morphology and contractile function of zebrafish larvae due to its high spatiotemporal resolution, minimal photodamage, and optical sectioning in contrast to other optical imaging methods.^16–19^ To better understand the myocardial structure and contractility during heart formation, development, injury, and repair, we developed a holistic strategy that integrates LSFM system construction and control, retrospective synchronization, individual cell tracking, and interactive VR platform for quantitative assessments. Overall, our framework enables us to efficiently image the heart, reconstruct the 4D model, track and quantify individual cells, providing an entry point to assess cardiomyocytes functionalities and regional myocardial deformation.

Our customized LSFM system provides a cellular resolution of 1.26 *μ*m in the lateral direction and 2.48 *μ*m in the axial. While the anisotropic resolution of our current LSFM might affect the quality of 4D reconstruction, the cellular resolution of this customized system is sufficient for distinguishing zebrafish cardiomyocytes with the diameter ranging from 10 to 20 *μ*m.^29,30^ The 3D reconstruction model of fluorescent beads further verifies the resolving power of our current system (supplementary material video 2). Further efforts in multi-view fusion and deconvolution techniques will enhance our imaging capabilities, allowing us to overcome the limitations stemming from resolution disparities between different imaging directions.^13^

Our LSFM system with retrospective reconstruction method allows us to analyze the contracting ventricle and atrium at over 200 volumes per second. However, the deluge of data in 4D light-sheet imaging remains a challenge in both acquisition and analysis. Our system generated approximately 100 GB of images in a single fluorescent channel within five cardiac cycles. Due to the independence of each Z-movie, parallel computation depending on multi-core CPU and GPU is able to improve the reconstruction efficiency over ten folds. However, parallel computing also poses challenges for memory management. To avoid memory fragmentation and computer slowdown caused by excessive memory consumption, we optimized our algorithm by minimizing the size of memory allocation and deallocation. For instance, we cleared the loaded images after each call and experimented with different types of image buffers. As a result, our memory usage increased much less than the reconstruction efficiency. We performed a quantitative comparison to demonstrate the improvement in memory performance of our parallel computing algorithm (supplementary material Fig. S3).

While the retrospective synchronization assumes that cardiomyocytes return to their original positions after each cardiac cycle, previous studies have demonstrated the feasibility of this method and indicated that the heartbeat variability in zebrafish embryos is only ∼1% in the period length between consecutive beats,^15,16^ echoed by our results in Figs. 5(d) and 5(e). Other methods such as light-field microscopy hold great potential to minimize the impact of this assumption and redundant sampling in a single slice.^31–33^ In addition, our results indicate a larger fluctuation of the SA:V ratio in the atrium in comparison to the ventricle [Fig. 6(g)], implying that the multi-view deconvolution approach could improve the image contrast if the atrium is away from the detection objective lens.^13^ To further improve the accuracy and efficiency of the reconstruction algorithm, we will also investigate two methods for identifying cardiac cycle and aligning cardiac phases: the sum of squared differences (SSD)^34^ and Pearson's correlation.^35^ The former one is potentially useful for finding cardiac cycle in each Z-movie as this approach assumes that all processed images have a similar range of intensities. The latter is better suited for cardiac phase alignment across different Z-movies as it assumes a linear relationship among all processing images.

While computational analysis of cardiac contractile function has been established in some imaging modalities,^21^ approaches tailored for LSFM are still under development. Due to the contribution of cardiomyocytes to cardiac contraction,^36,37^ we utilized the 3DeeCellTracker tool to track individual cardiomyocytes from end-systole to end-diastole. More training data for this neural network will definitely improve the tracking accuracy from 80% to a higher level, but a data-efficient method is also needed to reduce the time-consuming manual annotation. Our ongoing efforts in machine learning^38,39^ provide an entry point to minimize the training dataset for this purpose. By integrating LSFM and deep learning methods, we investigated representative cardiomyocytes in the atrium, ventricular apex, base, and atrioventricular canal of zebrafish at 3 and 7 dpf. Our findings indicate that ventricular myocardium undergoes less deformation, which could be attributed to the thicker myocardium of the ventricle.^40^ We also found that the cardiomyocytes at 3 dpf exhibited a larger displacement [Figs. 5(d) and 5(e)] and SA:V ratio [Fig. 6(g)] compared to those at 7 dpf. These findings suggest that myocardial properties or hemodynamics may contribute to the changes observed as the fish grow.^41,42^ However, it is crucial to emphasize that conducting a comprehensive statistical analysis is imperative for future investigations to further understand and validate these observations.

In addition to the current study of myocardial contractility using the transgenic *Tg(cmlc2:nucGFP)* zebrafish line, other transgenic models such as *Tg(tp1:GFP)* for Notch activity and *Tg(fli1:DsRed)* for endocardium could also extend our study in elucidating cardiac morphogenesis and regeneration (supplementary material Fig. S4). One possible extension of our method is to implement multi-channel fluorescence imaging to track numerous lineages and cell types such as cardiomyocytes, endothelial cells, and fibroblasts for the study of cellular heterogeneity and intercellular interaction during the cardiac development and regeneration.^43^ Ongoing efforts on the integration of advanced imaging methods such as light-field microscopy will enable us to investigate hemodynamics, hemostasis, and thrombosis, as well as their interaction with cardiac function in the live zebrafish models.^44–46^

To better visualize and analyze the 4D heart model, we also developed a VR platform for user-directed manipulation with more freedom. Our platform provides an immersive and interactive experience for studying individual cells and global patterns with high accuracy and efficiency. This enables us to quantify the cellular velocity and SA:V ratio change across the entire ventricle and atrium, investigate the physical relationship between arbitrary cells and regions during the contraction, and further deepen our understanding of their roles in myocardial mechanics.^41,47^ The use of VR will also enable us to perform complex tasks such as cell segmentation and annotation more efficiently and accurately, as users can interact with the data in a straightforward manner and adjust parameters in real time.^23–25^ Since this progressive research provides more potential for the study of intricate architecture and dynamics, we will improve its generalizability and functionality for more animal models across numerous scales.^13,24^

Different from conventional ejection fraction or strain analysis, our computational analysis allows us to unravel the difference in regional myocardial contractile function under physiological and pathophysiological conditions.^21,48^ Our continuous efforts in 4D live imaging enable us to investigate the underlying mechanisms of heart disease that perturb cardiac structure and function. The integration of our 4D imaging platform and multi-dimensional computation with transgenic zebrafish models provides an entry point for the *in vivo* investigation of myocardial infarction, cardiac arrhythmias, and congenital heart defects. With the enhancement of computation power, we will assess more cardiac phases and cycles to capture the variability of contractility over time. A more comprehensive assessment including stress and strain analysis will also advance our understanding of myocardial contractile function at both cellular and tissue levels. Collectively, we have proved that this holistic strategy allows for the *in vivo* study of cardiac contractile function, holding great promise to uncover the mechanism underlying cardiac morphogenesis and facilitate new therapies.

## METHODS

### Customized LSFM system with program control

We built an in-house LSFM imaging system (supplementary material Fig. S2) using a continuous-wave diode-pumped solid-state (DPSS) laser system with dual wavelengths at 473 nm (LRS-0473-GFM-00100–03, Laserglow Technologies) and 532 nm (LRS-0532-GFM-00100–03, Laserglow Technologies) as the illumination sources. The initial beam diameter was ∼2 mm, with a divergence less than 1.5 mrad. Both beams were aligned before passing through a 5× achromatic beam expander (GBE05-A, Thorlabs) and a variable iris (ID25, Thorlabs). The aligned beam was focused by a plano–convex cylindrical lens (*f* = 50 mm, Thorlabs) and relayed by a pair of lenses (CLS-SL, ITL200, Thorlabs) to the back focal plane of the illumination objective lens (Plan Fluor, 4×/0.13, Nikon). This optical setup yields a light sheet with a beam waist of 1 *μ*m and an effective width of 2 mm. The detection module was composed of a water-immersion objective lens (Plan Fluor, 20×/0.5, Olympus) for fluorescence detection, a tube lens (ITL180, Thorlabs), a filter set (Semrock), and an sCMOS camera (Flash 4.0 v3, Hamamatsu). A 3D-printed chamber was made to hold the zebrafish larva and the detection objective. A six-axis stage was installed to precisely position the specimen. The control of this whole system was customized via LabVIEW 2020 for laser illumination, sample scanning, and fluorescence detection.

### PSF calibration

We diluted the fluorescent beads to a concentration of 1:150 000 using a solution of 0.8% agarose with de-ionized water. The diluted beads were mounted in a fluorinated ethylene propylene (FEP) tube and immersed in a water-filled chamber for imaging. The waist of the Gaussian beam, determined by the excitation wavelength and the numerical aperture (NA) of the illumination objective lens dominates the axial resolution in the LSFM system. In our light-sheet setup, axial resolution is mainly given by the beam waist, and other factors include the NA of the detection lens, the refractive index, and the emission wavelength.^49^

### Preparation of the transgenic zebrafish sample

Transgenic *Tg(cmlc2: nucGFP)*, *Tg(tp1: GFP)*, and *Tg(fli1a: DsRed)* zebrafish lines were used. Animal protocols, experiments, and housing in this manuscript have been approved (IACUC #20–07) and conducted under the oversight of the University of Texas at Dallas and University of Texas at Arlington Institutional Animal Care and Use Committee. To maintain the optical transparency of the larvae, we added 0.003% phenylthiourea (PTU) in the medium to suppress pigmentation of larvae at 20 hours post fertilization (hpf). Zebrafish larvae were anesthetized in 0.05% tricaine for 10 min to ensure immobilization, followed by the immersion in 0.8% low-melt agarose at 37 °C in the FEP tube (refractive index: ∼1.33). The whole specimen with the FEP tube was held by the translational stages and placed inside the chamber filled with water. *Tg(cmlc2: nucGFP)* larvae were imaged every 24 h from 72 to 168 hpf, while *Tg(tp1: GFP)* and *Tg(fli1a: DsRed)* lines were imaged at 96 and 120 hpf.

### Image acquisition

The sCMOS camera continuously recorded Z-movies (image sequences), while the light-sheet sectioned a thin layer of the contracting heart at different depths. We reiterated the recording-scanning process to capture 300 frames in each Z-movie to cover 3–5 cardiac cycles. To meet the Nyquist-Shannon sampling theorem, we set the step size between two consecutive slices as 1 *μ*m and captured 100–200 Z-movies to cover the entire volume.

### Image registration

To register different Z-movies, the first key step was to estimate the cardiac cycle length. The number of images *N* between the first and fourth peak systolic frames in the first movie was manually counted, and the average cycle length L was defined as L = N/3 * E, where *E* is the exposure time. The cardiac cycle length was empirically between 0.85 L to 1.15 L.^17^ Then, each Z-movie was split into different cycles based on each possible cycle length from 0.85 L to 1.15 L, and a within-movie similarity comparison was performed for each Z-movie. Specifically, every image in each cycle was back projected to the previous cycle, and the SSD between the two cycles was calculated. The cycle length with the smallest SSD was chosen as the most possible cycle length. The second key step was to align the starting points of Z-movies at different axial locations. An iterative image similarity comparison through the first Z-movie to the last Z-movie was used. First, a clip including around three cardiac cycles in the first Z-movie with start slice number *S* was cropped. Multiple clips in the second and third Z-movies were cropped with the same length but with adjusted start slice number *S + D*, where *D* stands for the cardiac phase difference between these two Z-movies. To determine the best *D* for aligning the first two clips, we computed the SSD between them. We also calculated the SSD between the clips of the first and third Z-movies, as well as the second and third Z-movies, to avoid local optimization.^15^ We picked the correct clip in the second Z-movie by identifying the smallest SSD and aligned all subsequent Z-movies iteratively using the same method.

### Parallel computation

We used two parallelism strategies to speed up the processing of images in our algorithm. First, we implemented task parallelism into the CPU multi-core parallel processing by adapting the MATLAB parallel computing toolbox into our established algorithm.^50^ Next, we applied the data parallelism strategy to the image to be processed. We converted them into *gpuArray* datatype and used CUDA (Compute Unified Device Architecture) to perform computations on GPU. The improved reconstruction algorithm was programmed using MATLAB and tested on a workstation equipped with Intel i9-10900X CPU (3.7 GHz, 10 cores), 64.0 GB RAM, NVIDIA Quadro RTX 5000 graphics card (3072 CUDA cores, 16 GB GDDR6 memory).

### Cell tracking

We implemented the deep learning method, 3DeeCellTracker,^22^ to segment cardiomyocytes at different cardiac phases. We used watershed method^51^ to separate continuous regions of voxels, and then assigned each region a number. The segmented cells were manually corrected only in the first volume of 3D images. In the following 3D tracking step, feedforward network was used to predict cell positions based on spatial patterns of cells maintained between previous and current images. The predicted positions are corrected with PR-GLS^52^ to obtain precise cell locations. We visually inspected the tracking results by comparing the locations of tracked cells with the corresponding raw images.

### VR analysis for assessment of cardiac activities

Our VR analysis framework consisted of processing and segmenting zebrafish cardiac images, producing a surface mesh for each individual cardiomyocyte nucleus, model importing, and interaction with each individual nucleus to intuitively analyze the cardiac contractility and morphology such as cell trajectory during each cycle. To interact with the 4D heart model in the virtual environment, we started by obtaining the cell tracking results from 3DeeCellTracker. Next, we validated the tracking data and saved cells with consistent image intensity across all volumes in Python. Afterward, we customized a Python program to generate a surface mesh and assign a unique color code to each cell in 3D Slicer. The heart models including all cells in different timepoints were exported as *.obj* files with *.mtl* files and imported into Unity. We further customized C# programs to allow for 4D visualization and interactive analysis. The VR platform allows for cell selection, time point selection, time pause, and lighting adjustment. The user can select two cells and analyze their trajectories, velocities, volume, surface area, and relative distance. The time point selection option allows the user to choose a specific time for 4D data analysis.

## SUPPLEMENTARY MATERIAL

See the supplementary material, which includes four supplementary figures and nine supplementary videos. Supplementary material Fig. 1 provides the overview of the zebrafish cardiac imaging and analysis pipeline. Supplementary material Fig. 2 depicts illustrations and photos of our LSFM hardware system. Supplementary material Fig. 3 compares the workstation memory usage for 4D reconstruction algorithms. Supplementary material Fig. 4 includes other transgenic models including *Tg(tp1:GFP)* for Notch activity and *Tg(fli1:DsRed)* for endocardium under our LSFM microscope. All supplementary material videos provide supporting materials including the LSFM system simulation, the 4D reconstruction and cell tracking, and the VR analysis of the heart.

## Data Availability

The data that support the findings of this study are openly available in Zenodo at https://doi.org/10.5281/zenodo.7953887, Ref. 53.
